# Continuity of care of Swiss residents aged 50+: a longitudinal study using claims data

**DOI:** 10.1136/ihj-2021-000105

**Published:** 2022-03-24

**Authors:** Anna Nicolet, Isabelle Peytremann-Bridevaux, Joël Wagner, Clémence Perraudin, Christophe Bagnoud, Joachim Marti

**Affiliations:** 1 Center for Primary Care and Public Health (Unisanté), Lausanne, Switzerland; 2 University of Lausanne, Lausanne, Vaud, Switzerland; 3 Center for Primary Care and Public Health, Lausanne, Switzerland; 4 Department of Actuarial Science, Faculty of Business and Economics, University of Lausanne, Lausanne, Switzerland; 5 Swiss Finance Institute, University of Lausanne, Lausanne, Switzerland; 6 Groupe Mutuel, Martigny, Switzerland

**Keywords:** Continuity of patient care, Health services research, Longitudinal studies

## Abstract

**Background:**

Continuity of care (COC) should be measured for healthcare quality monitoring and evaluation and is a key process indicator for integrated care. Measurement of COC using routinely collected data is widespread, but there is no consensus on which indicator to use and the relevant time horizon to apply. Information about COC is especially warranted in highly fragmented healthcare systems, such as in Switzerland. Our study aimed to compare COC measures in Swiss residents aged 50+ obtained with various indices and time horizons.

**Methods:**

Using insurance claims data, we computed and compared several commonly used visit-based Continuity of Care Indices (COCIs): Bice-Boxerman Index, Usual Provider of Care, Herfindahl-Hirschman Index, Modified, Modified Continuity Index and Modified Continuity Index, based on all doctor visits and on primary care (PC) visits only. Indices were computed over short (1 year) and medium (4 years) terms.

**Results:**

The mean indices based on all visits varied between 0.51 and 0.77, while PC indices presented less variation with a median of 1.00 for all but one index. Indices focusing on a variety of individual providers decreased with time horizon, while indices focusing on the overall number of visits and providers showed the opposite trend. These findings suggest fundamental differences in the interpretation of COCIs.

**Conclusions:**

Broad COC appeared moderately low in Switzerland, although comparable to other countries, and PC COC was close to one. The choice of indices and time horizon influenced their interpretation. Understanding these differences is key to select the appropriate index for the monitoring of COC.

**Key messages**

**What is already known about this subject?**

- With rising multimorbidity, transition from traditional healthcare arrangements towards integrated care structures is needed.
- Continuity of care (COC) is viewed as a cornerstone in primary care and family medicine and is a key process indicator for integrated care.
- A wide variety of COC indicators measured over 1–2 years in claims data have been used in the health services research literature.

**What does this study add?**

- We investigate the effect of time horizon on COC, which was not broadly studied. Indices calculated over a longer period reflect COC differently from yearly indices, as they capture more potential variation in the visit pattern to healthcare providers.
- The choice of index is important to consider while measuring COC, whereby indices focused on contributions of individual healthcare providers (Continuity of Care Index, Usual Provider of Care and Herfindahl-Hirschman Index) should be distinguished from the indices focused on the overall amount of services and providers (Modified, Modified Continuity Index and Modified Continuity Index). The latter ones are more suitable as indicators of needs rather than COC.
- We show that broad COC appeared moderately low among Swiss residents, while COC with general practitioners appeared high in Switzerland.

**How might this impact on clinical practice or future developments?**

- Availability of routinely collected administrative data is increasing. Wider use of such data in countries without national registries may help the monitoring of COC.
- Our study might improve the rationale behind the choice of COC indicators and time horizon.
- Our study may help the design of future research aimed at assessing the impact of new care models on care continuity.

## Introduction

Continuity of care (COC) is a key process indicator for integrated care and should be monitored for healthcare quality monitoring and evaluation. It reflects regular visits to a health professional, sustained over time, a relationship of trust and responsibility between patients and health professionals.^1^ COC can be considered as a quality of care indicator, of particular importance for older adults with multiple chronic conditions, especially in fragmented health systems with free provider choice, lack of care coordination and primary care (PC) weaknesses such as Switzerland.^2^ Despite a large heterogeneity in the literature regarding COC indices, time horizon and data sources,^3^ claims data are widely used to measure longitudinal COC at the population level or in specific patient groups,^4–6^ with application of indices often ranging from 0 to 1, where one indicates highest possible COC. Such indices may reflect concentration (higher proportion of visits to a specific doctor among other doctors imply higher COC), dispersion (higher overall number of doctors visited imply lower COC), density (higher number of visits to a particular provider implies higher COC) or sequence of doctor visits (whether the same doctor is visited from one time to the next).^7^ Studies using claims data typically measure COC with various indices over 1–2 years,^3^ while applying a longer time frame could be appropriate to assess whether sustained high COC is associated with health outcomes.^4^ Although claims data collected over long observation periods for billing and reimbursement purposes allow monitoring over multiple years and are increasingly accessible,^8^ the effect of time horizon on COC was not broadly investigated. Moreover, the richness of claims data allows calculation of multiple indices and exploration of differences in their interpretations.

Our objective was to report on the COC of Swiss residents aged 50+ and to investigate the differences in COC measured by various indices and for different time horizons.

## Methods

We used data from Groupe Mutuel, one of the largest health insurance companies providing mandatory health insurance to 11.4% of the Swiss population. The data include information on more than 240'000 randomly selected, continuously enrolled individuals aged 50+ in 2015–2018, representing 70% of the whole pool of insured in this age group in Groupe Mutuel. In addition to basic demographic variables, the data included detailed information on reimbursed physician visits, including specialty, and a unique provider identifier for each visit. The data did not include information on visits to other health professionals, such as nurses, physiotherapists, etc, which allowed us to measure COC only within physicians. We computed several commonly used visit-based COC indices (accounting all consultations (broad) vs PC only)^3 7^ (table 1) and present descriptive statistics in 2015 and over 4 years. In order to quantify longitudinal COC, particularly meaningful for more complex patients with multiple visits to healthcare providers, we calculated the indices only for the subgroup of individuals with multiple visits per year (>2).^6^ This subgroup might therefore have slightly higher needs than the average 50+ population.

**Table 1:** Calculation and types of COC indices

Index formulas All indices assign a value between 0 and 1 to each patient, with 1 indicating highest possible COC.	Type of COC	1-year COC Longitudinal COC is meaningful for patients with multiple visits.	4-year COC The minimum number of visits was lowered to avoid a large loss of observation.
If *M* denotes the total number of providers, *N* the total number of visits, and *n_i_*. the number of visits to provider *i*: 1. Bice-Boxerman Continuity of cCare Index= $\frac{\sum_{i=1}^{M}{\text{n}}_{\text{i}}²-N}{N\left(N-1\right)}$ . 2. Usual Provider of Care= $\frac{max\;n_{i}}{N}$ . 3. Herfindahl-Hirschman Index= $\sum_{i=1}^{M}\left(\frac{{\text{n}}_{i}}{N}\right)²$ . 4. Modified Continuity Index= $1-\frac{M}{N+0.1}$ . 5. Modified, Modified Continuity Index = $\frac{1-\frac{M}{N+0.1}}{1-\frac{1}{N+0.1}}$ .	Broad COC	Calculated for individuals with three and more visits per year (n>2), based on all contacts with physicians across all specialties*	Calculated for individuals with nine and more visits over 4 years (n>10), based on all contacts with physicians across all specialties
	PC-COC	Calculated for individuals with three and more visits per year (n>2), based on visits to general practitioners only	Calculated for individuals with nine and more visits over 4 years (n>10), based on visits to general practitioners only

*That is, general internal medicine, allergology and clinical immunology, anesthesiology, angiology, cardiology, surgery, dermatology and venereology, endocrinology and diabetology, gastroenterology, gynaecology and obstetrics, physical medicine and rehabilitation, neurology, medical oncology, ophthalmology, otorhinolaryngology, pathology, pulmonology, psychiatry and psychotherapy, radiation oncology and radiotherapy, radiology, rheumatology and urology.

COC, continuity of care; PC, primary care.

## Results

The sample consisted of 240'419 enrollees in 2015 (table 2). Participants had on average 5.6 visits to the general practitioners and 4.4 visits to specialists. The mean broad Continuity of Care Indices (COCIs) varied between 0.51 (Continuity of Care Index (COCI)) and 0.77 (Modified, Modified Continuity Index (MMCI)). The mean PC-COCIs presented less variation (0.81 to 0.95) with a median of 1.00 for all but one index. Broad and PC indices calculated over 4 years were lower than indices calculated in 2015, except for Modified Continuity Index (MCI) and MMCI.

**Table 2:** Characteristics of the sample and COC indices

Characteristics of the sample, n (%)	2015	Over 4 years
Total sample size	240'419	239'986
Number of deaths (%)	–	2.02
Age (years), mean, (SD)	63.90 (10.41)	66.90 (10.41)
Men (%)	47.99	47.99
Model with gatekeeping (%)	49.88	54.92

COC indices	2015		Over 4 years	
	Mean (95% CI)	Med (IQR)	Mean (95% CI)	Med (IQR)
Broad-UPC	0.67 (0.67 to 0.67)	0.67 (0.50–0.86)	0.58 (0.58 to 0.58)	0.57 (0.42–0.74)
PC-UPC	0.94 (0.94 to 0.94)	1.00 (0.94–1.00)	0.87 (0.87 to 0.87)	0.95 (0.79–1.00)
Broad-COCI	0.51 (0.51 to 0.51)	0.46 (0.29–0.71)	0.40 (0.40 to 0.40)	0.36 (0.24–0.53)
PC-COCI	0.89 (0.89 to 0.90)	1.00 (0.89–1.00)	0.79 (0.79 to 0.79)	0.88 (0.61–1.00)
Broad MCI	0.68 (0.68 to 0.68)	0.72 (0.59–0.81)	0.81 (0.81 to 0.81)	0.83 (0.75–0.89)
PC-MCI	0.81 (0.81 to 0.81)	0.84 (0.76–0.90)	0.92 (0.92 to 0.92)	0.93 (0.90–0.96)
Broad MMCI	0.77 (0.77 to 0.77)	0.80 (0.67–0.91)	0.83 (0.83 to 0.83)	0.85 (0.78–0.91)
PC-MMCI	0.95 (0.95 to 0.95)	1.00 (0.96–1.00)	0.96 (0.96 to 0.96)	0.97 (0.93–1.00)
Broad HH	0.57 (0.57 to 0.57)	0.52 (0.36–0.74)	0.42 (0.42 to 0.42)	0.38 (0.26–0.54)
PC-HH	0.91 (0.91 to 0.91)	1.00 (0.90–1.00)	0.80 (0.80 to 0.80)	0.88 (0.63–1.00)
Healthcare use				
Total number of consultations	10.00 (9.95 to 10.04)	7.00 (2.00–14.00)	43.44 (43.28 to 43.61)	33.00 (15.00–60.00)
Number of consultations with general practitioner	5.62 (5.59 to 5.65)	3.00 (0.00–8.00)	24.23 (24.13 to 24.34)	17.00 (5.00–34.00)
Number of consultations with specialists	4.38 (4.35 to 4.41)	2.00 (0.00–6.00)	19.20 (19.09 to 19.31)	11.00 (3.00–25.00)
Number of specialist doctors visited	1.69 (1.68 to 1.70)	1.00 (0.00–3.00)	5.07 (5.06 to 5.11)	4.00 (2.00–7.00)

Categorical variables were described by relative frequencies, continuous variables by means with 95% CIs and medians with IQRs.

COC, continuity of care; COCI, Continuity of Care Index; HH, Herfindahl-Hirschman Index; MCI, Modified Continuity Index; MMCI, Modified, Modified Continuity Index; PC, primary care; UPC, Usual Provider of Care.

## Discussion

Our study showed that broad COC appeared moderately low among Swiss residents, although comparable to other countries^9 10^; PC-COC was particularly high. A recent study among patients with cancer in Switzerland^5^ corroborated our findings of high COC with general practitioners in Switzerland, and that MMCI was consistently higher than other indices. These results highlight that the Swiss healthcare system relies traditionally on PC: most patients tend to visit one general practitioner even in a system without generalised gatekeeping. This high PC-COC could be better exploited to develop more integrated care strategies in Switzerland, and therefore improve care efficiency and quality.

Indices differed in their interpretation (likely due to calculation formulas), which explains the impact the time horizon had on COC. Measures focusing on the share of visits to specific providers (COCI, Usual Provider of Care and Herfindahl-Hirschman Index) decreased over time: the number of providers and visits increase over time due to higher healthcare needs, decreasing individual provider shares, resulting in lower indices. Conversely, indices focusing on the total number of providers and visits (MCI and MMCI) increased with time horizon as the number of visits cumulates at a larger pace than the number of different doctors. Therefore, MCI and MMCI are more likely to express needs rather than COC. The choice of time horizon is therefore key when COC is monitored. For a complex, multimorbid population (with deteriorating health), indices calculated on the longer term better capture potential variation in visit patterns. Additionally, with the increasing appeal for integrated team-based and interprofessional models of care, there is a need to develop indices that go beyond conventional approaches based on physician visits.

Our study results should be interpreted considering the general limitations of claims data. First, we lack information about appropriateness of care. COC measures close to 1 may not correspond to appropriate care if a patient needed other health professional visits than the general practitioner. Second, the number of visits may be underestimated, as visits to health professionals other than physicians and non-covered visits by the insurance company could not be captured.

Researchers need to carefully consider reliability and the interpretations behind existing indices before measuring COC in a particular population group and time horizon, or consider developing more advanced novel measures reflecting COC within integrated healthcare structures, different from the conventionally assumed relation between a single provider and a patient. Future research could benefit from investigating the impact of COC measured in different time horizons on health and economic outcomes. Furthermore, understanding the drivers of COC and identifying population subgroups with low COC are important for broader policy implications.
